# Green Spaces, Land Cover, Street Trees and Hypertension in the Megacity of São Paulo

**DOI:** 10.3390/ijerph17030725

**Published:** 2020-01-22

**Authors:** Tiana C. L. Moreira, Jefferson L. Polizel, Itamar de Souza Santos, Demóstenes F. Silva Filho, Isabela Bensenor, Paulo A. Lotufo, Thais Mauad

**Affiliations:** 1 Faculdade de Medicina, Departamento de Patologia, Universidade de São Paulo, São Paulo 01246-903, Brazil; tmauad@usp.br; 2 Harvard T. H. CHAN School of Public Health, Environmental Health Department, Boston, MA 02215, USA; 3 Escola Superior de Agricultura “Luiz de Queiroz”, Universidade de São Paulo, Piracicaba 13418-900, Brazil; jlpolize@usp.br (J.L.P.); dfilho@usp.br (D.F.S.F.); 4 Centro de Pesquisa Clínica, Hospital Universitário, Universidade de São Paulo, São Paulo 05508-000, Brazil; itamarss@usp.br (I.d.S.S.); isabensenor@gmail.com (I.B.); palotufo@usp.br (P.A.L.); 5 Faculdade de Medicina, Departamento de Clínica Médica, Universidade de São Paulo, São Paulo 01246-903, Brazil; 6 Instituto de Estudos Avançados, Universidade de São Paulo, São Paulo 05508-060, Brazil

**Keywords:** cardiovascular health, constructed area, high resolution images, São Paulo megacity

## Abstract

Proximity to green spaces has been shown to be beneficial to several cardiovascular outcomes in urban spaces. Few studies, however, have analyzed the relationship between these outcomes and green space or land cover uses in low–medium income megacities, where the consequences of rapid and inordinate urbanization impose several health hazards. This study used a subgroup of the dataset from The Brazilian Longitudinal Study of Adult Health ELSA-BRASIL (*n* = 3418) to identify the correlation between the medical diagnosis of hypertension and green spaces in the megacity of São Paulo. Land cover classification was performed based on the random forest algorithm using geometrically corrected aerial photography (orthophoto). Three different indicators of exposure to green spaces were used: number of street trees, land cover and number of parks within 1 km. We used logistic regression models to obtain the association of the metrics exposure and health outcomes. The number of street trees in the regional governments (OR = 0.937 and number of parks within 1 km (OR = 0.876) were inversely associated with a diagnosis of hypertension. Sixty-three percent of the population had no parks within 1 km of their residence. Our data indicate the need to encourage large-scale street tree planting and increase the number of qualified parks in megacities.

## 1. Introduction

The rapid concentration of populations in large urban agglomerations has produced megacities, i.e., cities with more than 10 million inhabitants. A range of potential health hazards are present in megacities, especially in the developing world: traffic-related air pollution, substandard housing, urban violence, vector-borne diseases, inadequate sanitation, and insufficient or contaminated drinking water, among others [1]. São Paulo city, the 10th most populated megacity in the world, has more than 11 million habitants [2] and its metropolitan area is home to 21 million individuals. 

The abovementioned adverse impacts on the health of a megacity can be mitigated by the availability of green spaces, which can offer different ecosystem services (ESSs) and create beneficial health effects. The ESSs associated with green spaces fall into different categories: provisioning (e.g., food, water, fuel and wood); regulating (e.g., regulation of temperature, water, disease); cultural (e.g., educational, aesthetic, recreation) and supporting services (e.g., primary production and soil formation) [3]. 

Several studies have associated the beneficial effects of green space proximity with major health-adverse outcomes [4,5,6]. These beneficial effects on health stem from factors including a reduction in air pollutants, noise, and wind impact [7,8], decrease in temperatures [9,10], and promotion of physical activities and social cohesion [11]. An additional mechanism through which green spaces may enhance human health is through stress reduction, which may in turn reduce blood pressure [12,13,14]. Authors recommend that, in cities, 1–10 ha of green space should be accessible within 5 min walk of the residence, i.e., 300–500 m [15]. 

The beneficial outcomes from green space on cardiovascular health have been previously demonstrated in studies [16,17,18,19]. In Boston, proximity to green areas was associated with higher rates of survival after an ischemic stroke [16]. A positive effect was also observed in Taipei, where green structures were associated with lower cardiovascular mortality rates, mediated by the reduction in air pollution and heat [17].

However, few studies have addressed cardiovascular parameters related to land cover; most studies analyzed only the density of green areas [20,21]. There is increasing evidence that exposure to street trees has a significant beneficial effect on human health [22,23,24], possibly linked to their capacity to filter air pollution. Finally, the beneficial use of parks for cardiovascular health has been shown to be mediated by physical activity, social cohesion and the ecosystemic benefits of green areas [19]. The effects of green areas and land cover on health in megacities of low–middle income countries like Sao Paulo have been poorly studied [25,26]. 

In this study, we aimed to evaluate the association between the number of street trees, land cover, and distance from parks with the diagnosis of hypertension in a cross-sectional analysis of the Brazilian Longitudinal Study of Adult Health (ELSA-BRASIL) participants living in São Paulo. ELSA-BRASIL is a large cohort study focusing on the cardiovascular outcomes of civil servants aged 35 to 74 years in six Brazilian cities; the prevalence of hypertension in this population is 35.8% [27]. Due to the urban complexities of a megacity, we decided to explore a 300 m buffer and larger areas such as districts and regional governmental boundaries.

## 2. Materials and Methods 

This study was approved by the Research Ethics Committee of the School of Medicine, São Paulo, Brazil (number: 64262016.3.0000.0065).

### 2.1. Study Population

The main focus of ELSA-BRASIL is on the incidence and risk factors for cardiovascular diseases and diabetes in Brazilian adults. ELSA-BRASIL is a cohort study of 15,105 civil servants living in six cities, as previously described [28,29]. In this study, we analyzed a sample of the civil servant population from the University of São Paulo living in São Paulo. This subgroup had 3418 participants that were enrolled in the period 2008–2010.

As social demographic characteristics, we used gender, age, educational level, race, income, smoking habits, body mass index, and level of physical activity, while the selected cardiovascular risk factors were diabetes, dyslipidemia, excessive drinking and salt consumption, as described above.

The educational level was ascertained in years and classified in levels (less than high school, high school and college or above). Race was self-reported as black, mixed, white, or other (Asian or native indigenous). Smoking status was self-reported as never, past, or current. Body mass index (BMI) was defined as weight (kilograms) divided by squared height (meters). Physical activity levels were obtained using the leisure and commuting domains of the International Physical Activity Questionnaire—IPAQ [30]—and defined as ideal (≥75 min/week of vigorous physical activity, or ≥150 min/week of moderate physical activity or ≥150 min/week of moderate + vigorous physical activity); intermediate (1–149 min/week of moderate + vigorous activity); or poor (no moderate + vigorous physical activity); as adopted in other analyses [31].

Diabetes was defined as previous diabetes diagnosis, the use of medication to treat diabetes, fasting plasma glucose (FPG; ≥126 mg/dL; ≥7.0 mmol/L), 2-h plasma glucose during the OGTT (Oral Glucose Tolerance Test)—2 h PG ≥ 200 mg/dL; ≥11.1 mmol/L, or HbA1C (Hemoglobin A1c or glycated hemoglobin)—≥6.5%; ≥47.5 mmol/mol [29].

Dyslipidemia was defined as the reported use of lipid-lowering treatments or low-density lipoprotein cholesterol level ≥3.36 mmol/L (130 mg/dL). Excessive drinking data were obtained in the interviews and defined as >210 g alcohol/week for men and >140 g alcohol/week for women [29]. Salt intake was estimated from 24-h urine sodium excretion samples [32]. Seven different categories were used according to sodium excretion: 1 (<3 g); 2 (3–5.9 g); 3 (6–8.9 g); 4 (9–11.9 g); 5 (12–14.9 g); and 6 (15–17.9 g) 7 (>18 g).

### 2.2. Hypertension Definition

The information on medical and laboratory assessments of ELSA-BRASIL participants can be found in Aquino et al. (2012), Schmidt et al. (2013) and Bensenor et al. (2013) [28,33,34]. In the ELSA-BRASIL, hypertension was defined as a systolic BP ≥140 mmHg or diastolic BP ≥90 mmHg or the reported use of high blood pressure medication.

### 2.3. Study Area

This study was conducted in São Paulo city, which currently has 12.25 million inhabitants [35]. São Paulo’s urban explosion, which created a megacity, occurred in the 1950s with the development of the vehicle industry and increasing industrialization along the highways, which attracted migrants from other parts of Brazil. The large influx of people caused a housing crisis, and the city fringe became populated without urban planning, good transportation or primary services. This irregular expansion affected important agricultural and wooded areas, with several irregular settlements in those areas [36]. Currently, there is high disparity in the distribution of green areas in the city, and only five regional governmental boundaries within the city have more than 15 m^2^ of green area per habitant [37]. Traffic-related air pollution in São Paulo city, including PM_2.5_ levels, consistently exceeds the levels recommended by the World Health Organization [38] in São Paulo.

**Figure 1:**
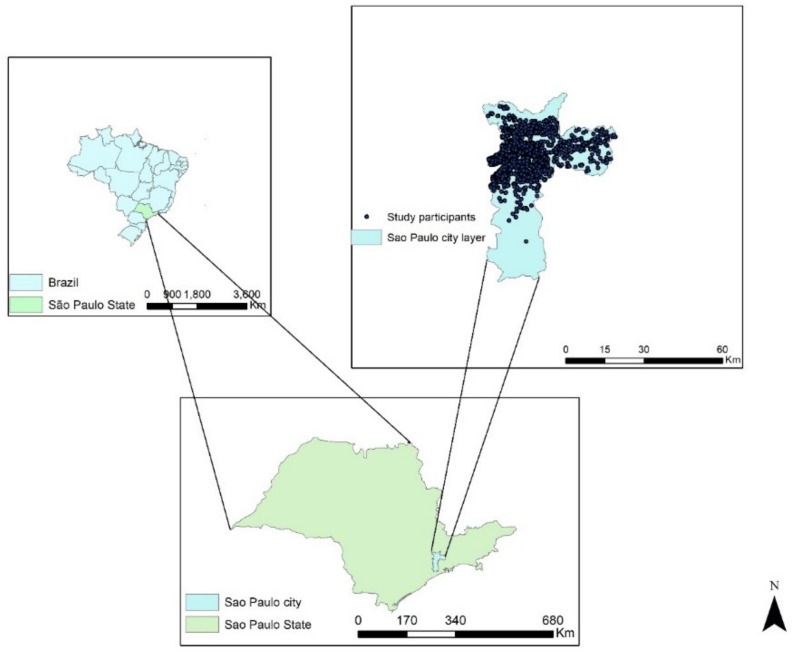
São Paulo city layer, Brazil and the distribution of the Longitudinal Study of Adult Health (ELSA-BRASIL) participants within the city (*n* = 3418).

The residential distribution of ELSA-BRASIL participants in São Paulo and the São Paulo city layer are shown in Figure 1.

### 2.4. Street Trees Map

A digital map of street tree locations in São Paulo was provided by the municipality of São Paulo. The map identified urban trees on sidewalks, street islands and roundabouts and excluded trees in squares, parks, reserves, and internal public and private areas. The map was generated by quantitative photos and local inventory analysis of trees (orthophoto of 2010). The images had a 2 m resolution on a 1:25,000 scale [39].

### 2.5. Land Cover Classification

**Figure 2:**
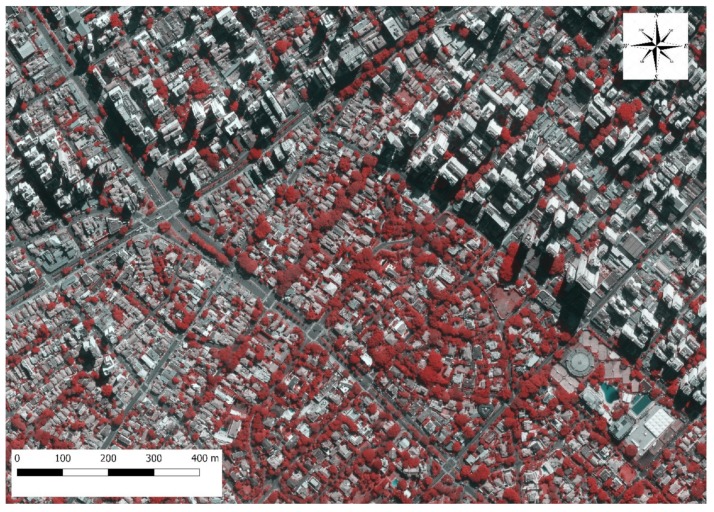
Sample of the orthophoto (2-m spatial resolution) of São Paulo with the near infrared band of QGIS2.18.11, showing green areas in shades of red.

A São Paulo city orthophoto (Figure 2) from 2010, provided by the São Paulo Institute of Geography and Cartography, was used for supervised classification.

The orthophoto had a spatial resolution of 2 m on the pixel side with 3 spectral bands: near infrared (NIR), blue and red. The land cover classification was performed using the random forest algorithm (program QGIS2.18.11; Plugin Dtezaka). Random forest (RF) is a powerful learning classifier algorithm that is one of the most accurate methods of classifying land cover [40]. The RF is a general term for ensemble methods that use tree-type classifiers to train the random forest algorithm, which creates multiple CART-like trees [41]. For classification, each tree in the RF chooses a unit vote for the most popular class (pixel color) at the data input (trainer samples polygon = data input). The output of the classifier is determined by a majority vote of the class [42]. For the trainer samples, the classification of the classes was performed according to pixel color and spectral signature. One hundred and fifty training samples were prepared for each class of land cover. The images were classified within the following land cover classes: tree canopy, grass, bare soil, cement floor, swimming pool, shade, roof (white, gray, dark, ceramic), asphalt and river/lake (adapted from Myeong et al., 2003). For data analysis, we considered the sum of tree canopies and grass as green space and the sum of the different types of roof as constructed areas.

We used a false color composite scheme to allow for simple vegetation detection in the image. In this type of false color composite image, vegetation appears in different shades of red depending on vegetation type and condition due to its high reflectance in the NIR band [43]. Bare soil, roads and buildings may appear in various shades of blue, yellow or gray, depending on their material composition. The orthophoto false color composition used in this study was R (channel 1) = NIR band, G (channel 2) = red band, B (channel 3) = blue band.

The classification accuracy was determined using an error matrix, the Kappa index, which indicates the presence of misclassification. The thematic maps used in this study had Kappa values equal to or above 81%, which is considered an accurate classification according to [44].

### 2.6. Urban Parks

Using QGis 2.18.11, we drew a map of the São Paulo urban parks using the shapefile of the polygons of the municipal parks at the portal GeoSampa [39] and the state parks in the São Paulo orthophoto.

### 2.7. Land Cover Exposure Assessment

**Figure 3:**
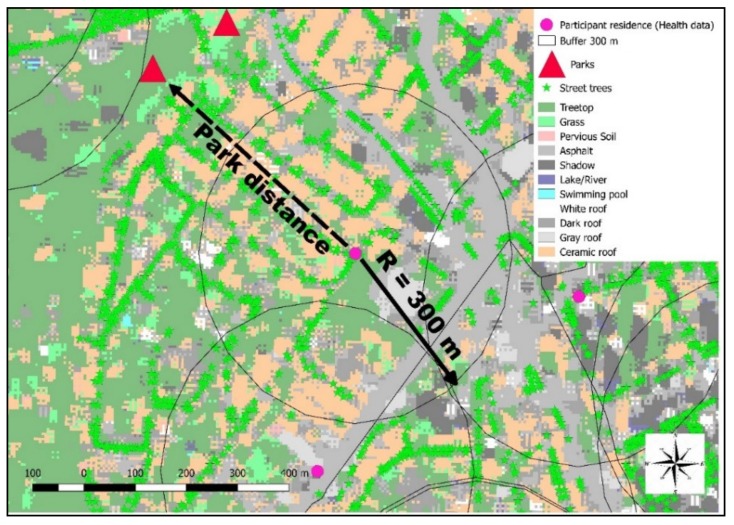
Exposure assessment metrics (Examples of the 300 m buffers and Euclidean distance of parks).

Three different green space exposure indicators were used: number of street trees, land cover and Euclidian distance (Figure 3) from urban parks (reserves excluded) using the program QGIS2.18.11.

**Figure 4:**
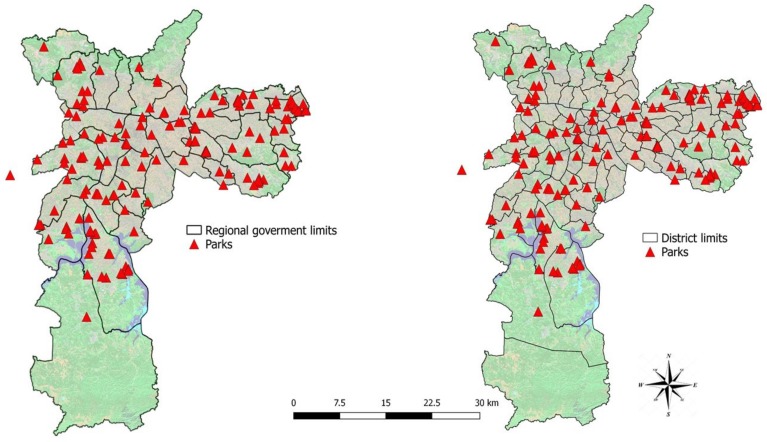
District and regional government boundaries exposure assessment and park distribution.

Street tree databases and land cover were evaluated within 300 m radius buffers (Figure 3) around each residence of the ELSA-BRASIL participants. The WHO document “Green Spaces and Health” [45] recommended the 300 m buffer, as it corresponds to approximately 5 min walking distance along walkable roads or pathways. In addition, the number of street trees and amount of land cover were assessed within the 96 districts and 32 São Paulo regional government boundaries. (Figure 4). In São Paulo, regional governments are responsible for, among other tasks, the maintenance of the urban green areas within their limits. Each regional government boundary encompasses a certain number of districts that share similar geographic, urban, demographic and economic features [46].

From the residence of each participant, we measured the Euclidian distance to the 3 closest parks and counted the number of parks within a 1 km Euclidian distance (0–3 parks). The number of parks was also used as a categorical variable (0 park, 1 park, 2 parks and 3 parks).

### 2.8. Data Analysis

R 3.4.1 software for Windows was used for the statistical analysis. For each buffer, the district and regional government boundary data were presented as the mean (±SD), minimum and maximum percentage of the number of street trees, all land cover classes and the mean (±SD) distance of parks.

We performed logistic regression analyses to identify the association between street trees, land cover and the number of parks within 1 km of the participant’s residence and the diagnosis of hypertension. Street trees and land cover data represented the 300 m buffer, district boundaries and regional government boundaries. The variance inflation factor (VIF) was used to check the multicollinearity of the variables, and binary logistic regression was used for dichotomous dependent variables. The significance level was set at *p*  =  0.05.

The data are presented as odds ratios (95% confidence intervals). The models were adjusted for sociodemographic variables (age, gender, education level, and race) and cardiovascular risk factors (smoking habits, BMI, diabetes, dyslipidemia, excessive drinking, salt consumption and level of physical activity) from ELSA-BRASIL.

A sensitivity analysis using the individual incomes extracted from the ELSA-BRASIL database and the neighborhood Human Development Index (HDI) [47] was performed by a regression model to check whether the neighborhood socioeconomic status could have interfered in the results. 

## 3. Results

The sample from ELSA-Brasil in this study (*n* = 3418) presented a mean age of 52.3 (SD = 9.2); 56% of the sample were women, 62.1% were white; and 32% were hypertensive.

The sociodemographic characteristics (Table 1) and the associated cardiovascular risk factors of the study population are presented (Table 2). The risk factors for cardiovascular diseases considered in ELSA-BRASIL were smoking habits, BMI, physical activity, excessive drinking, hypertension, diabetes, dyslipidemia and salt consumption for the study population (*n* = 3418).

We did not find any significant correlation between the number of street trees and any land cover classes (tree canopy, grass, green areas, bare soil, river/lake, swimming pools and roofs) and hypertension diagnosis within the 300 m buffer.

The Descriptive statistic of land cover classes of 300 m buffers, districts and regional government boundaries (Table S1) and the descriptive statistic of proximity (meters) of tree closest park (*n* = 3418) (Table S2) can be found on the supplementary material. 

The percentage of tree canopy and green space within the districts presented a tendency toward negative odds ratios (OR). There was a positive effect on the OR for hypertension diagnosis for each 1% of the constructed area within the districts; the associated OR was 1.011. There was no association between street trees and hypertension in the districts.

The number of street trees in the regional government boundaries was significantly associated with a diagnosis of hypertension in this population (Table 3). An increase of 10,000 street trees in the regional government boundaries was associated with an OR = 0.937. There was no association between all land cover classes and hypertension in the regional government boundaries.

The distribution of the study population according to the number of parks within 1 km was 63.5% (zero parks), 26.2% (one park), 6.4% (two parks) and 3.9% (three parks). No participant had more than three parks within a 1 km Euclidian distance. Having more than one park within 1 km of the participant residence was associated with an OR = 0.876. Additionally, the OR for the presence of two parks was 0.74 (*p* = 0.04; CI (95%): 0.532 to 0.986). No associations were found with the presence of three parks because of the low frequency of individuals with this condition (3.9%).

The VIF did not show multicollinearity between the variables used in the regression models.

The descriptive statistics and the prevalence of hypertension in the different districts are presented in the supplementary material.

The sensitivity analysis did not show significant effects of individual incomes or neighborhood socioeconomic status (assessed by HDI) on the diagnosis of hypertension for those parameters with significance in the previous regression models (Tables S3 and S4).

## 4. Discussion

In this study, we have shown that the number of street trees within the local regional government boundaries and the presence of parks within 1 km of the residence have a negative association with hypertension diagnosis in an adult population living in the megacity of São Paulo. In addition, our data showed a positive association with the proportion of constructed areas and hypertension diagnosis in this sample of ELSA-BRASIL.

Our data showed that an increase of 10,000 trees in the regional government boundaries was associated with a hypertension diagnosis OR of 0.93. Several studies have demonstrated positive associations between street trees and different health outcomes in larger cities [23,24,48]. Reid et al. (2017) found associations between better self-reported health and the number of street trees (but not grass) within a 1000 m buffer in New York [48]. Lovasi et al. (2008) showed that an increase in tree density was associated with a lower asthma prevalence in children in New York City [24]. Another study in the same city [23] correlated street trees and birth outcomes and described a significant inverse association between nearby street trees and the odds of preterm birth for all women. That study did not identify a consistent significant relationship between adverse birth outcomes and the normalized difference vegetation index (NDVI), access to major green spaces, or waterfront access adjusted for individual covariates.

The mechanisms by which street trees modulate the consistent positive associations with health are not clear. There is a known association between vehicular air pollution and cardiovascular morbidity and mortality [49]. Street trees are able to remove vehicular air pollution through dry deposition [50], thereby decreasing household exposure. On the other hand, green spaces also might be related to allergic respiratory diseases due to pollens and the release of volatile organic compounds that contribute to increasing air pollution [51,52,53]. In addition, the beneficial effects of green spaces on hypertension might be diminished by living close to major roads [54]. Interestingly, in the ELSA-BRASIL population, there was a positive association between commuting-related physical activity and hypertension among women, which could be explained by greater air pollution exposure [55]. According to Donovan (2017), a tree in areas with higher air pollution levels should have a greater impact on human health than the same tree in areas with lower levels of air pollution [56].

We observed a very irregular distribution of green areas and street trees within the urban fabric of São Paulo, resulting in clear disparities in access to green spaces, with extremes ranging from three to 899 trees within the 300 m buffer. Our data also showed that 63% of this population did not have any parks within 1 km of their residence, and 62% were considered poorly physically active. Distance to green spaces is considered one of the more important factors related to physical activity and green space use [57,58]. Some studies have demonstrated that beyond a 300–400 m distance to the individuals’ residence, the use of green spaces begins to decline very quickly [59,60].

Our results showed that people living in areas with more than one park within a 1 km distance of their residence had a lower OR (0.87) for hypertension diagnosis, with an OR of 0.72 for the individuals who had two parks within a 1 km distance of the residence. Several ESSs provided by green areas could be related to this finding: a better local microclimate with a lower temperature and a reduction in air pollution provided by the park, increased physical activity and reduction in stress [61]. Green space exposure is associated with less blood cortisol, which in turn is associated with chronic stress and is a risk factor for cardiovascular diseases [62]. In our study, we lack information on the use of parks for the practice of physical activity, but previous data suggest a beneficial synergistic effect of park distance and use [63] in relation to the incidence of fatal cardiovascular disease.

The % of constructed areas (sum of all classes of roofs) had a weak positive association with hypertension diagnosis in this study, with an OR of 1.01. There are some studies suggesting an increased prevalence of hypertension in urban areas when compared to rural areas [64]. Few studies, however, have analyzed different levels of constructed areas in relation to cardiovascular outcomes. A study from Taiwan found that those living in areas with the highest level of urbanization had the highest prevalence of strokes [65]. The mechanisms involved in this correlation could be related to higher air pollution, temperature levels and stress/crowding in more urbanized areas.

Interestingly, we did not observe health associations using the 300 m buffer for all the land cover classes or street trees, but associations were found using larger scale areas, such as the regional government boundaries. In New York, Reid et al. (2017) did not find positive effects of green spaces on self-reported health using a 300 m buffer [48]. Given the complexities of megacity urbanization, with high density of constructions and several environmental negative pressures, it is possible that the health-associated ecosystem services provided by green spaces are not felt within a 300 m buffer.

We could not find significant associations between the proportion of total green areas (grass + tree canopy) and hypertension diagnosis in the 300 m buffer, districts and regional government boundaries. There are a few possible explanations for these results. It is possible that green areas in São Paulo are not sufficient in their density, biodiversity or quality, such that the ESSs provided by these areas are not as expected. Fear of violence in nonqualified green areas may hinder their use by the population, showing that green spaces should not be considered equal in terms of the ESS offered [66]. Accordingly, a study using the dataset of the Amsterdam Health Survey (2004) linked the data of neighborhood stressors with cardiovascular outcomes in different races (divided into groups) and observed that high quality green space was associated with lower odds of hypertension, but only in the Moroccan group of the study [67]. It is possible that, if locally unwanted land uses or land uses perceived as unsafe were transformed in qualified green areas, we could have observed different results. 

Our study is one of the few studies that investigates the relationships between multiple urban land cover exposure measures and health outcomes in a megacity. Previous studies addressing the correlation of green space and health outcomes used green space metrics such as the NDVI [68,69,70], green space [71] or tree cover [72] around residential locations. Given the urban complexity of a megacity such as São Paulo, we believe the methods we have used provide a comprehensive approach to capturing the different land covers of the city. Another strength of this work is the excellent quality of the health data obtained by the ELSA study.

Our study also has its limitations. We could not obtain information in the present study about the access, quality, usability, and maintenance status of the parks and green areas in this study, which could have improved our data. In addition, we do not have information about eventual disservices of green spaces such as pollen and volatile organic compound emissions in the city of São Paulo. Although there are large socioeconomic disparities in São Paulo that could have influenced our data, the studied population (civil servants) had a better socioeconomic status than the average for the Brazilian population. This fact is also a possible explanation for the lack of a significant associations between individual incomes and neighborhood HDI in the adjusted regression models. Another important limitation of this study is its cross-sectional nature, which does not allow us to determine causation. It would be interesting to perform similar studies in other megacities to compare the main findings.

## 5. Conclusions

Even in cities with low-density and irregularly distributed green areas, living in regions with more street trees and nearby parks has a beneficial impact on the diagnosis of hypertension. Our results encourage large-scale street tree planting and the creation of more qualified parks to make cities healthier, especially in megacities. This study aimed to sensitize decision makers by providing information on the number of benefits of green spaces and their associated positive impacts on human health.

## Figures and Tables

**Table 1:** Study population sociodemographic characteristics (*n* = 3418).

Socioeconomic Variables	Groups	Results
**Gender**	Man	44.0%
	Woman	56.0%
**Age (years)**	Mean	52.3
	Range	35–74
	SD *	±9.2
**Educational level**	Lower than high school	12.4%
	High school	34.1%
	College or above	41.9%
**Income**	Low income—<USD1245	25.0%
	High income—≥USD3320	32.0%
	Medium income—USD1245–3319	41.9%
**Race**	White	62.1%
	Mixed	18.8%
	Black	11.3%
	Other	6.3%

* Standard deviation.

**Table 2:** The risk factors for cardiovascular diseases considered in ELSA-BRASIL (*n* = 3418).

Risk Factor Variables	Groups	Results
**Smoking habits**	Current smoker	16.06%
	Past smoker	30.84%
	Never smoked	53.10%
**Body mass index (BMI)**	Mean	27.10
	SD	±4.85
**Physical activity**	Ideal	26.07%
	Intermediate	12.08%
	Poor	61.85%
**Excessive drink**	No	94.55%
	Yes	5.45%
**Hypertension**	No	67.47%
	Yes	32.53%
**Diabetes**	No	76.67%
	Yes	20.33%
**Dyslipidemia**	No	42.05%
	Yes	57.95%
**Salt consumption (g) According to sodium excretion**	<3 g	2.01%
	3–5.9 g	14.86%
	6–8.9 g	25.23%
	9–11.9 g	22.16%
	12–14.9 g	16.11%
	15–17.9 g	8.75%
	>18 g	10.88%

**Table 3:** Odds ratios (and 95% confidence intervals) for the association between hypertension diagnosis, land cover and green space variables.

Variable	Crude	Model 1 Adjusted	Model 2 Adjusted
	OR (CI)	OR (CI)	OR (CI)
Street trees (300 m buffer)	1.037 (0.984 to 1.092)	1.068 (1.010 to 1.131) *	1.059 (0.996 to 1.126) ”
Street trees (distric)	0.980 (0.937 to 1.024)	0.988 (0.940 to 1.038)	0.988 (0.937 to 1.043)
Street trees (government)	0.922 (0.875 to 1.024) **	0.929 (0.878 to 0.984) *	0.937 (0.881 to 0.996) *
Green space (300 m buffer)	0.998 (0.922 to 1.004)	0.998 (0.992 to 1.004)	0.999 (0.992 to 1.006)
Green space(distric)	0.993 (0.987 to 1.000) ”	0.992 (0.984 to 0.999)	0.993 (0.985 to 1.001) ”
Green space (government)	0.991 (0.981 to 1.002)	0.992 (0.980 to 1.003)	0.991 (0.978 to 1.003)
Tree canopy (300 m buffer)	0.998 (0.991 to 1.005)	0.998 (0.991 to 1.005)	1.000 (0.992 to 1.008)
Tree canopy (distric)	0.990 (0.981 to 0.999) *	0.988 (0.978 to 0.997) *	0.990 (0.980 to 1.000) ”
Grass (300 m buffer)	0.994 (0.976 to 1.014)	0.993 (0.972 to 1.014)	0.993 (0.971 to 1.015)
Grass (distric)	1.000 (0.977 to 1.022)	0.994 (0.970 to 1.018)	0.992 (0.967 to 1.019)
Roofs (300 m buffer)	1.005 (0.999 to 1.011) ”	1.004 (0.997 to 1.011)	1.004 (0.997 to 1.011)
Roofs (distric)	1.010 (1.001 to 1.081) *	1.012 (1.003 to 1.021) **	1.011 (1.002 to 1.021) *
Parks less than 1 km	0.921 (0.826 to 1.027)	0.902 (0.801 to 1.015)	0.876 (0.769 to 0.998) *
1 park within 1 km	0.940 (0.797 to 1.108)	0.943 (0.789 to 1.126)	0.930 (0.766 to 1.127)
2 parks within 1 km	0828 (0.641 to 1.069)	0.763 (0.577 to 1.009) ”	0.724 (0.532 to 0.986) *
3 parks within 1 km	1.002 (0.090 to 1.077	1.021 (0.952 to 5.061)	0.000 (0.000 to 0.000)

Model 1 is adjusted for age, sex, race and educational level. Model 2 is adjusted for age, sex, race, educational level, smoking habits, body mass index, excessive drinking, salt consumption, physical activity, dyslipidemia diagnoses and diabetes diagnoses. Signif. codes: ** *p* < 0.01; * *p* < 0.05; ” *p* < 0.1.

## Supplementary Materials

The following are available online at https://www.mdpi.com/1660-4601/17/3/725/s1, Table S1: Descriptive statistics—Mean (%), standard deviation (%SD), minimum (%) e maximum (%) land cover classes of 300 m buffers, districts and regional government boundaries, Table S2: Descriptive statistic of proximity (meters) of tree closest park (*n* = 3418), Table S3: Sensibility analyses (regression models). Odds ratios (and 95% confidence intervals) for the association between hypertension diagnosis, land cover and green space variables using individual income groups. Table S4: Sensibility analyses (regression models). Odds ratios (and 95% confidence intervals) for the association between hypertension diagnosis, land cover and green space variables using HDI government boundaries groups.

## References

[1] McMichael A.J. (2000). The urban environment and health in a world of increasing globalization: Issues for developing countries. Bull. World Health Organ..

[2] IBGE Conheça Cidades e Estados do Brasil. www.cidades.ibge.gov.br.

[3] Millennium Ecosystem Assessment Board (2005). Ecosystems and Human Well-Being: Wetlands and Water Synthesis.

[4] Nieuwenhuijsen M.J., Khreis H., Triguero-Mas M., Gascon M., Dadvand P. (2017). Fifty shades of green: Pathway to healthy urban living. Epidemiology.

[5] Gascon M., Triguero-Mas M., Martínez D., Dadvand P., Rojas-Rueda D., Plasència A., Nieuwenhuijsen M.J. (2016). Residential green spaces and mortality: A systematic review. Environ. Int..

[6] James P., Hart J.E., Banay R.F., Laden F. (2016). Exposure to greenness and mortality in a nationwide prospective cohort study of women. Environ. Health Perspect..

[7] Nowak D.J., Dwyer J.F. (2007). Understanding the Benefits and Costs of Urban Forest Ecosystems.

[8] Fang C.-F., Ling D.-L. (2005). Guidance for noise reduction provided by tree belts. Landsc. Urban Plan..

[9] Oliveira S., Andrade H., Vaz T. (2011). The cooling effect of green spaces as a contribution to the mitigation of urban heat: A case study in Lisbon. Build. Environ..

[10] Solecki W.D., Rosenzweig C., Parshall L., Pope G., Clark M., Cox J., Wiencke M. (2005). Mitigation of the heat island effect in urban New Jersey. Glob. Environ. Chang. Part. B Environ. Hazards.

[11] Ngom R., Gosselin P., Blais C., Rochette L. (2016). Type and proximity of green spaces are important for preventing cardiovascular morbidity and diabetes—A cross-sectional study for Quebec, Canada. Int. J. Environ. Res. Public Health.

[12] Araya R., Montgomery A., Rojas G., Fritsch R., Solis J., Signorelli A., Lewis G. (2007). Common mental disorders and the built environment in Santiago, Chile. Br. J. Psychiatr..

[13] Astell-Burt T., Mitchell R., Hartig T. (2014). The association between green space and mental health varies across the lifecourse. A longitudinal study. J. Epidemiol. Community Health.

[14] Van den Berg A.E., Maas J., Verheij R.A., Groenewegen P.P. (2010). Green space as a buffer between stressful life events and health. Soc. Sci. Med..

[15] Senetra A., Krzywnicka I., Mielke M. (2018). An analysis of the spatial distribution, influence and quality of urban green space—A case study of the Polish city of Tczew. Bull. Geogr. Socio Econ. Ser..

[16] Wilker E.H., Wu C.-D., McNeely E., Mostofsky E., Spengler J., Wellenius G.A., Mittleman M.A. (2014). Green space and mortality following ischemic stroke. Environ. Res..

[17] Shen Y.-S., Lung S.-C.C. (2016). Can green structure reduce the mortality of cardiovascular diseases?. Sci. Total Environ..

[18] Dzhambov A.M., Markevych I., Lercher P. (2018). Greenspace seems protective of both high and low blood pressure among residents of an Alpine valley. Environ. Int..

[19] Yeager R.A., Smith T.R., Bhatnagar A. (2019). Green environments and cardiovascular health. Trends Cardiovasc. Med..

[20] Pereira G., Foster S., Martin K., Christian H., Boruff B.J., Knuiman M., Giles-Corti B. (2012). The association between neighborhood greenness and cardiovascular disease: An observational study. BMC Public Health.

[21] Lanki T., Siponen T., Ojala A., Korpela K., Pennanen A., Tiittanen P., Tsunetsugu Y., Kagawa T., Tyrväinen L. (2017). Acute effects of visits to urban green environments on cardiovascular physiology in women: A field experiment. Environ. Res..

[22] Kardan O., Gozdyra P., Misic B., Moola F., Palmer L.J., Paus T., Berman M.G. (2015). Neighborhood greenspace and health in a large urban center. Sci. Rep..

[23] Abelt K., McLafferty S. (2017). Green streets: Urban green and birth outcomes. Int. J. Environ. Res. Public Health.

[24] Lovasi G.S., Quinn J.W., Neckerman K.M., Perzanowski M.S., Rundle A. (2008). Children living in areas with more street trees have lower prevalence of asthma. J. Epidemiol. Community Health.

[25] Mowafi M., Khadr Z., Bennett G., Hill A., Kawachi I., Subramanian S.V. (2012). Is access to neighborhood green space associated with BMI among Egyptians? A multilevel study of Cairo neighborhoods. Health Place.

[26] Jia X., Yu Y., Xia W., Masri S., Sami M., Hu Z., Yu Z., Wu J. (2018). Cardiovascular diseases in middle aged and older adults in China: The joint effects and mediation of different types of physical exercise and neighborhood greenness and walkability. Environ. Res..

[27] Chor D., Pinho Ribeiro A.L., Sá Carvalho M., Duncan B.B., Andrade Lotufo P., Araújo Nobre A., de Aquino E.M.L.L., Schmidt M.I., Griep R.H., Molina M.D.C.B. (2015). Prevalence, awareness, treatment and influence of socioeconomic variables on control of high blood pressure: Results of the ELSA-Brasil study. PLoS ONE.

[28] Aquino E.M.L., Barreto S.M., Bensenor I.M., Carvalho M.S., Chor D., Duncan B.B., Lotufo P.A., Mill J.G., Molina M.D.C., Mota E.L.A. (2012). Brazilian longitudinal study of adult health (ELSA-Brasil): Objectives and design. Am. J. Epidemiol..

[29] Schmidt M.I., Hoffmann J.F., de Fátima Sander Diniz M., Lotufo P.A., Griep R.H., Bensenor I.M., Mill J.G., Barreto S.M., Aquino E.M.L., Duncan B.B. (2014). High prevalence of diabetes and intermediate hyperglycemia—The Brazilian longitudinal study of adult health (ELSA-Brasil). Diabetol. Metab. Syndr..

[30] Physical Activity Guidelines Advisory Committee (2008). Physical Activity Guidelines Advisory Committee Report.

[31] Machado L.B.M., Silva B.L.S., Garcia A.P., Oliveira R.A.M., Barreto S.M., Fonseca M., de Jesus M., Lotufo P.A., Bensenor I.M., Santos I.S. (2018). Ideal cardiovascular health score at the ELSA-Brasil baseline and its association with sociodemographic characteristics. Int. J. Cardiol..

[32] Mill J.G., da Silva A.B.T., Baldo M.P., Molina M.C.B., Rodrigues S.L. (2012). Correlation between sodium and potassium excretion in 24- and 12-h urine samples. Braz. J. Med. Biol. Res..

[33] Bensenor I.M., Griep R.H., Pinto K.A., de Faria C.P., Felisbino-Mendes M., Caetano E.I., da Silva Albuquerque L., Schmidt M.I., Bensenor I.M., Griep R.H. (2013). Rotinas de organização de exames e entrevistas no centro de investigação ELSA-Brasil. Revista de Saúde Pública.

[34] Schmidt M.I., Griep R.H., Passos V.M., Luft V.C., Goulart A.C., de Souza Menezes G.M., Molina M.D.C.B., Vigo A., Nunes M.A. (2013). Strategies and development of quality assurance and control in the ELSA-Brasil. Revista de Saude Publica.

[35] IBGE População. https://cidades.ibge.gov.br/brasil/sp/sao-paulo/panorama.

[36] Silva L.S.E. (2013). A Cidade e a Floresta: O impacto da Expansão Urbana Sobre Áreas Vegetadas na Região Metropolitana de São Paulo (RMSP). Ph.D. Thesis.

[37] Amato-Lourenço L.F., Moreira T.C.L., de Arantes B.L., da Silva Filho D.F., Mauad T. (2016). Metrópoles, cobertura vegetal, áreas verdes e saúde. Estudos Avançados.

[38] De Miranda R.M., de Fatima Andrade M., Fornaro A., Astolfo R., de Andre P.A., Saldiva P. (2012). Urban air pollution: A representative survey of PM2.5 mass concentrations in six Brazilian cities. Air Qual. Atmos. Health.

[39] São Paulo City Government Sistema de Consulta do Mapa Digital da Cidade de São Paulo. http://geosampa.prefeitura.sp.gov.br/.

[40] Rodriguez-Galiano V.F., Ghimire B., Rogan J., Chica-Olmo M., Rigol-Sanchez J.P. (2012). An assessment of the effectiveness of a random forest classifier for land-cover classification. ISPRS J. Photogramm. Remote Sens..

[41] Breiman L. (2001). Random forests. Mach. Learn..

[42] Gislason P.O., Benediktsson J.A., Sveinsson J.R. (2006). Random forests for land cover classification. Pattern Recognit. Lett..

[43] Jackson R.D., Huete A.R. (1991). Interpreting vegetation indices. Prev. Vet. Med..

[44] Landis J.R., Koch G.G. (1977). An application of hierarchical kappa-type statistics in the assessment of majority agreement among multiple observers. Biometrics.

[45] World Health Organization (2016). Urban Green Spaces and Health. A Review of Evidence.

[46] São Paulo City Government Conheça um Pouco mais das Secretaria Municipal das Prefeituras Regionais da Cidade de São Paulo. http://www.prefeitura.sp.gov.br/cidade/secretarias/regionais/subprefeituras/index.php?p=8978.

[47] Marguti O.B., Aurélio C.M., Favarão C.B. (2017). Territórios em números: Insumos para políticas públicas a partir da análise do IDHM e do IVS de UHDs e regiões metropolitanas brasileiras, livro 2.

[48] Reid C., Clougherty J., Shmool J., Kubzansky L. (2017). Is All urban green space the same? A comparison of the health benefits of trees and grass in New York City. Int. J. Environ. Res. Public Health.

[49] Brook R.D.M., Rajagopalan S., Pope C.A.I., Brook J.R., Bhatnagar A., Diez-Roux A.V., Holguin F., Hong Y., Luepker R.V.M., Mittleman M.A.M. (2010). Particulate matter air pollution and cardiovascular disease: An update to the scientific statement from the American Heart Association. Circulation.

[50] Maher B.A., Ahmed I.A.M., Davison B., Karloukovski V., Clarke R. (2013). Impact of roadside tree lines on indoor concentrations of traffic-derived particulate matter. Environ. Sci. Technol..

[51] Erbas B., Jazayeri M., Lambert K.A., Katelaris C.H., Prendergast L.A., Tham R., Parrodi M.J., Davies J., Newbigin E., Abramson M.J. (2018). Outdoor pollen is a trigger of child and adolescent asthma emergency department presentations: A systematic review and meta-analysis. Allergy.

[52] Lambert K.A., Lodge C., Lowe A.J., Prendergast L.A., Thomas P.S., Bennett C.M., Abramson M.J., Dharmage S.C., Erbas B. (2019). Pollen exposure at birth and adolescent lung function, and modification by residential greenness. Allergy.

[53] Silveira C., Tchepel O. Multiscale analysis of satellite-derived vegetation parameters for biogenic VOC emission modeling. Proceedings of the First International Conference on Remote Sensing and Geoinformation of the Environment.

[54] Brazienė A., Venclovienė J., Tamošiūnas A., Dėdelė A., Lukšienė D., Radišauskas R. (2018). The influence of proximity to city parks and major roads on the development of arterial hypertension. Scand. J. Public Health.

[55] Treff C., Benseñor I.M., Lotufo P.A. (2017). Leisure-time and commuting physical activity and high blood pressure: The Brazilian longitudinal study of adult health (ELSA-Brasil). J. Hum. Hypertens..

[56] Donovan G.H. (2017). Including public-health benefits of trees in urban-forestry decision making. Urban For. Urban Green..

[57] Cohen D.A., McKenzie T.L., Sehgal A., Williamson S., Golinelli D., Lurie N. (2007). Contribution of public parks to physical activity. Am. J. Public Health.

[58] Bjork J., Albin M., Grahn P., Jacobsson H., Ardo J., Wadbro J., Ostergren P.-O., Skarback E. (2008). Recreational values of the natural environment in relation to neighbourhood satisfaction, physical activity, obesity and wellbeing. J. Epidemiol. Community Health.

[59] Giles-Corti B., Broomhall M.H., Knuiman M., Collins C., Douglas K., Ng K., Lange A., Donovan R.J. (2005). Increasing walking: How important is distance to, attractiveness, and size of public open space?. Am. J. Prev. Med..

[60] Nielsen T.S., Hansen K.B. (2007). Do green areas affect health? Results from a Danish survey on the use of green areas and health indicators. Health Place.

[61] Akpinar A. (2016). How is quality of urban green spaces associated with physical activity and health?. Urban. For. Urban. Green..

[62] Ward Thompson C., Roe J., Aspinall P., Mitchell R., Clow A., Miller D. (2012). More green space is linked to less stress in deprived communities: Evidence from salivary cortisol patterns. Landsc. Urban. Plan..

[63] Tamosiunas A., Grazuleviciene R., Luksiene D., Dedele A., Reklaitiene R., Baceviciene M., Vencloviene J., Bernotiene G., Radisauskas R., Malinauskiene V. (2014). Accessibility and use of urban green spaces, and cardiovascular health: Findings from a Kaunas cohort study. Environ. Health.

[64] Wu Y., Huxley R., Li L., Anna V., Xie G., Yao C., Woodward M., Li X., Chalmers J., Gao R. (2008). Prevalence, awareness, treatment, and control of hypertension in China: Data from the China national nutrition and health survey 2002. Circulation.

[65] Lin H.-C., Lin Y.-J., Liu T.-C., Chen C.-S., Chiu W.-T. (2007). Urbanization and stroke prevalence in Taiwan: Analysis of a nationwide survey. J. Urban Health.

[66] Vieira J., Matos P., Mexia T., Silva P., Lopes N., Freitas C., Correia O., Santos-Reis M., Branquinho C., Pinho P. (2018). Green spaces are not all the same for the provision of air purification and climate regulation services: The case of urban parks. Environ. Res..

[67] Agyemang C., Hooijdonk C.V., Wendel-Vos W., Lindeman E., Stronks K., Droomers M. (2007). The association of neighbourhood psychosocial stressors and self-rated health in Amsterdam, the Netherlands. J. Epidemiol. Community Health.

[68] Dadvand P., Sunyer J., Basagana X., Ballester F., Lertxundi A., Fernandez-Somoano A., Estarlich M., Garcia-Esteban R., Mendez M.A., Nieuwenhuijsen M.J. (2012). Surrounding greenness and pregnancy outcomes in four Spanish birth cohorts. Environ. Health Perspect..

[69] Hystad P., Davies H.W., Frank L., Van Loon J., Gehring U., Tamburic L., Brauer M. (2014). Residential greenness and birth outcomes: Evaluating the influence of spatially correlated built-environment factors. Environ. Health Perspect..

[70] James P., Banay R.F., Hart J.E., Laden F. (2015). A review of the health benefits of greenness. Curr. Epidemiol. Rep..

[71] Maas J., Verheij R.A., Groenewegen P.P., De Vries S., Spreeuwenberg P. (2006). Green space, urbanity, and health: How strong is the relation?. J. Epidemiol. Community Health.

[72] Donovan G.H., Butry D.T., Michael Y.L., Prestemon J.P., Liebhold A.M., Gatziolis D., Mao M.Y. (2013). The relationship between trees and human health: Evidence from the spread of the emerald ash borer. Am. J. Prev. Med..

