# Supplementary material for: Green Spaces, Land Cover, Street Trees and Hypertension in the Megacity of São Paulo
Source: Int J Environ Res Public Health. 2020 Jan 22;17(3):725. doi: 10.3390/ijerph17030725 (PMC7038323; doi:10.3390/ijerph17030725)
Supplement: Supplementary file 1 [file ijerph-17-00725-s001.pdf]

Supplementary material

Elsa-Brasil Cohort description

The Longitudinal Study of Adult Health - ELSA Brazil - is a multicenter cohort investigation composed of 15,000 employees from six public higher education and research institutions in the Northeast, South and Southeast of Brazil. The aim is to investigate the incidence and risk factors for chronic diseases, in particular cardiovascular diseases and diabetes.

In each center of the study, the studied population - aged 35 to 74 years - take exams and interviews about as living conditions, social differences, and work relationship, gender and diet habits. In addition to fostering the development of new investigations, the study will be fundamental for adapting public health policies to national needs. The RMSP study has 5061 volunteers aged 35 to 74 years old who are all employees of the University of São Paulo, followed since 2008. The ELSA coordination in the state of São Paulo is under the responsibility of Paulo Andrade Lotufo and Vice-Coordinator of Isabela Judith Martins Benseñor. In São Paulo, the ELSA Research Center (CI - SP) is located at the University Hospital of the University of São Paulo (USP), located at Av. Lineu Prestes, 2565 - University City - ZIP Code 05508-900 - São Paulo - SP, see http://www.elsa.org.br/objetivos.html.

More information can be found at:

- Aquino, E. M., Barreto, S. M., Bensenor, I. M., Carvalho, M. S., Chor, D., Duncan, B. B., ... & Azeredo Passos, V. M. (2012). Brazilian longitudinal study of adult health (ELSA-Brasil): objectives and design. American journal of epidemiology, 175(4), 315-324.
- Bastos, M. S., Lotufo, P. A., Whitaker, A. L., & Bensenor, I. M. (2012). Validation of the short-version of Rose Angina Questionnaire in Brazil. Arquivos brasileiros de cardiologia, 99(5), 1056-1059.
- Lotufo, P. A. (2013). Setting up the longitudinal study for adult health (ELSA-Brasil). Revista de saude publica, 47, 3-9.
- Schmidt, M. I., Duncan, B. B., Mill, J. G., Lotufo, P. A., Chor, D., Barreto, S. M., ... & Carvalho, M. S. (2014). Cohort profile: longitudinal study of adult health (ELSA-Brasil). International journal of epidemiology, 44(1), 68-75.

Results

Descriptive statistics (means, SDs, percentages) were firstly calculated to describe and summarize the variables used in this study. Table S1 shows the proportions of land cover variables and number of street trees in the 300m buffers, 96 city district and 32 regional government boundaries.

Table S1. descriptive statistics – Mean (%), standard deviation (%SD), minimum (%) e maximum (%) land cover classes of 300 m buffers, districts and regional government boundaries

		Street trees (n)	Treetop (%)	Grass (%)	Green spaces (%)	Soil (%)	River/lake (%)	Swiming pool (%)	White roof (%)	Dark roof (%)	Gray roof (%)	Ceramic roof (%)	Roofs (%)
**300 m Buffer**	**Mean**	267	21.87	3.69	26.07	0.66	0.04	0.00	1.31	15.02	3.31	22.79	42.80
	**SD**	137	10.60	3.81	12.47	0.74	1.25	0.18	1.30	8.97	3.89	8.40	12.08
	**Minimum**	3	0.00	0.00	0.01	0.00	0.00	0.00	0.00	0.01	0.00	0.01	0.02
	**Maximum**	899	74.01	64.27	83.17	18.42	34.52	8.09	3.55	72.70	46.09	50.41	85.08
**District (n = 96)**	**Mean**	14317	23.49	6.52	30.01	0.38	1.78	0.17	1.44	18.69	2.42	20.40	42.95
	**SD**	16231	11.62	5.82	17.44	0.33	4.35	0.65	0.70	10.17	2.45	6.18	13.58
	**Minimum**	1290	4.97	0.95	5.92	0.01	0.01	0.00	0.11	0.44	0.30	7.46	10.69
	**Maximum**	59475	56.83	27.52	84.35	1.47	22.75	5.90	4.06	42.35	17.57	35.72	67.91
**Regional goverment (n=32)**	**Mean**	39508	23.68	4.98	28.66	8.52	4.60	0.33	1.84	15.91	2.49	3.74	23.98
	**SD**	13609	16.52	2.85	19.37	1.79	2.81	0.70	0.68	5.64	0.77	1.40	6.12
	**Minimum**	3520	6.20	2.00	8.20	4.03	1.43	0.06	0.81	2.20	0.77	2.00	9.08
	**Maximum**	88721	69.60	13.18	82.79	11.98	14.61	3.01	3.55	25.74	4.13	6.25	34.03

To analyze park distance, we considered the 3 parks (Table S2) closest from the participants' homes. The mean distance to the closest park was 1294.86 meters and the mean distance to the 3rd closest park was 2389 m. the participant living closest from the park is 37.07 m away and the thither is 11695.85 m.

Table S2. Descriptive statistic of proximity (meters) of tree closest park (N = 3418)

	Proximity from participant residence (m)		
	Park 1	Park 2	Park 3
Mean	1294.86	1909.57	2389
SD	717.22	905.07	944.53
Minimum	37.07	137.09	141.86
Maximum	6571.22	11400.4	11695.85

Table S3. Sensibility analyses (regression models). Odds ratios (and 95% confidence intervals) for the association between hypertension diagnosis, land cover and green space variables using individual income groups.

		**Crude**	**Model 1 adjusted**	**Model 2 adjusted**
	**Individual income**	OD (CI)	OD (CI)	OD (CI)
Street trees government boundaries	low income	**0.86 (0.79-0.95)****	**0.87 (0.79-0.97)***	0.90 (0.81 - 1.01)
	0.980 (0.937 to 1.024)	0.94 (0.87-1.02)	0.95 (0.87 - 1.03)	0.93 (0.85 - 1.02)
	high income	0.95 (0.86-1.05)	0.95 (0.85 - 1.06)	0.94 (0.84 - 1.06)
Parks less 1 Km	low income	0.84 (0.68 - 1.04)	0.79 0.63 - 1.00	0.83 0.65 - 1.07
	medium income	1.01 (0.85 - 1.20)	1.02 0.84 1.22	0.96 0.78 - 1.18
	high income	0.86 (0.71 - 1.04)	0.88 0.72 1.07	0.86 (0.69 - 1.07)
Roofs	low income	**1.01 (1.00 - 1.02)***	1.00 (0.99 - 1.02)	1.00 (0.99 - 1.02)
	medium income	1.00 0.99 - 1.01	0.99 (0.98 - 1.00)	0.99 (0.98 - 1.00)
	high income	1.00 (0.99 - 1.01)	1.00 (0.99 - 1.02)	1.01 (0.99 - 1.02)

Model 1 is adjusted for age, sex, race and educational level. Model 2 is adjusted for age, sex, race, educational level, smoking habits, body mass index, excessive drinking, salt consumption, physical activity, dyslipidemia diagnoses and diabetes diagnoses. Signif. codes: '***' 0.001 '**' 0.01 '*' 0.05 ' " ' 0.1.

Table S4. Sensibility analyses (regression models). Odds ratios (and 95% confidence intervals) for the association between hypertension diagnosis, land cover and green space variables using HDI government boundaries groups.

		**Crude**	**Model 1 adjusted**	**Model 2 adjusted**
	**IDH**	OD (CI)	OD (CI)	OD (CI)
Street trees government boundaries	> 0.800 (High)	0.98 (0.91 - 1.04)	0.97 (0.90 - 1.05)	0.96 (0.89 - 1.04)
	< 0.800 (Medium)	0.86 (0.69 - 1.07)	0.81 (0.64 - 1.03)	0.83 (0.65 - 1.07)
Parks less 1 Km	> 0.800 (High)	0.91 (0.81 - 1.03)	0.90 (0.79 - 1.03)	0.89 (0.77 - 1.02)
	< 0.800 (Medium)	1.03 (0.79 - 1.34)	0.94 (0.70 - 1.26)	0.97 (0.71 - 1.32)
Roofs	> 0.800 (High)	0.99 (0.98 - 1.00)	0.99 (0.98 - 1.00)	0.99 (0.98 - 1.00)
	< 0.800 (Medium)	**1.01 (1.00 - 1.02)***	1.00 (0.99 - 1.02)	1.01 (0.99 - 1.02)

Model 1 is adjusted for age, sex, race and educational level. Model 2 is adjusted for age, sex, race, educational level, smoking habits, body mass index, excessive drinking, salt consumption, physical activity, dyslipidemia diagnoses and diabetes diagnoses. Signif. codes: '***' 0.001 '**' 0.01 '*' 0.05 ' " ' 0.1.
